# Predicting 7-Year Survival in Individuals with Chronic Spinal Cord Injury: The Role of Self-Reported Functional Independence, Secondary Health Conditions, Comorbidities, and Health-Related Well-Being

**DOI:** 10.1159/ned/adwag018

**Published:** 2026-08-27

**Authors:** A. Feller, A. Buzzell, I. Eriks-Hoogland, M. Hund, X. Jordan, M. Schubert, M.W.G. Brinkhof

**Affiliations:** a Swiss Paraplegic Research, Nottwil, Switzerland; b Department of Health Sciences and Health Medicine, University of Lucerne, Lucerne, Switzerland; c Swiss Paraplegic Center, Nottwil, Switzerland; d REHAB Basel, Basel, Switzerland; e Clinique Romand de Réadaption, Sion, Switzerland; f Spinal Cord Injury Center, Balgrist University Hospital, Zurich, Switzerland

**Keywords:** Spinal cord injury, Survival, Self-reports, Population-based cohort study, Community setting

## Abstract

**Introduction:**

Previous research investigated prognostic factors for survival in prevalent cases of chronic spinal cord injury (CSCI) based on self-reported information. However, most of these studies originated in the USA and relied on cohorts drawn from individual hospitals or hospital networks. This population-based cohort study aims to identify prognostic factors affecting survival within the Swiss Spinal Cord Injury (SwiSCI) community, emphasizing self-reported data on functional independence (FI), secondary health conditions (SHCs), comorbidities, and emotional well-being.

**Methods:**

Data from the Swiss 2012 SCI community survey were linked to vital status information. Missing data were addressed using multiple imputation. FI was measured using the self-report version of the Spinal Cord Independence Measure (SCIM-III-SR). SHC and dimensions of health-related well-being were assessed using multi-categorical variables. Emotional well-being was evaluated using the Short-Form Health-Survey (SF-36). Continuous and multi-categorical predictors were dichotomized by selecting the most predictive cut-off. The prognostic model was developed using a flexible parametric survival model, with model selection guided by the Akaike Information Criterion, following the reduction of potential predictors based on univariable analyses. Basic demographic and lesion characteristics were included in all models under consideration (base model).

**Results:**

The study included 1,011 individuals with a median time since injury of 11 years. During the observation period, 110 deaths occurred. The model selection procedure identified the following self-reported predictors: SCIM-respiratory and sphincter management (HR: 1.77, *p* = 0.012), SCIM-self-care (HR 1.66, *p* = 0.038), contractures (HR; 1.42, *p* = 0.125), respiratory problems (HR;2.06, *p* = 0.002), and cancer (HR: 1.86, *p* = 0.0.084). From the base model, being male (HR: 2.15, *p* = 0.001) and age at survey (HR: 1.09, *p* < 0.001) were the strongest predictors. Four distinct model-based risk groups were established. The prognostic index demonstrated a good fit between observed and predicted survival, along with high discrimination between risk groups.

**Conclusion:**

Self-reported FI and health conditions, assessed years after injury, provide valuable prognostic information for the survival of prevalent cases with CSCI. However, internal and external validation of the existing models is required before clinical implementation can be considered.

## Introduction

Chronic spinal cord injury (CSCI) profoundly affects the lives of individuals and leads to significant physical, emotional, and social challenges [1, 2]. CSCI refers to a permanent or progressive disruption of nerve function in the spinal cord that persists for at least 1 year [3]. CSCI can arise from various causes, including trauma, disease, or degeneration, and typically leads to ongoing management and rehabilitation needs.

The life expectancy of individuals with CSCI is generally lower than that of the general population and is significantly affected by secondary health complications and the unique challenges of long-term disability management [4, 5]. Factors associated with life expectancy after spinal cord injury (SCI)/CSCI include the level and severity of the injury, age at the time of injury, sex, preexisting health conditions, secondary health conditions (SHCs), access to specialized care, and lifestyle choices [4, 5]. While short-term survival of individuals with SCI has improved in high-income countries, improvements in long-term survival have been observed in some studies but not in others [4, 5]. However, as the management of CSCI improves, the need for effective prognostic tools is becoming increasingly critical to understand patient outcomes and guide lifelong care. This is particularly important, as studies suggest that much of the excess mortality in CSCI is related to potentially treatable and/or preventable conditions such as respiratory and urinary tract infections [6–9]. Identifying survival probabilities can guide clinical decision-making and enhance outpatient management.

Recent studies have highlighted the importance of self-reported measures in understanding the health-related quality of life (QoL) and functional outcomes in individuals with CSCI [10–12]. Self-reports not only capture the subjective experiences of patients, but also provide valuable insights into their health status, resilience, and coping mechanisms. Furthermore, self-reports have proven to be valuable tools for assessing various health outcomes, including subsequent risk of mortality [13–15]. They are often easier and quicker to collect, enabling remote monitoring and timely interventions, which has been proven to lead to improved health outcomes and greater patient satisfaction [16, 17].

In Switzerland, interdisciplinary follow-up care appointments are scheduled in specialized SCI clinics at 3, 6, and 12 months post-discharge, continuing annually thereafter. However, a recent Swiss study revealed significant nonadherence to follow-up care, with 18% of patients failing to attend their first-year appointments [18]. The consequences of nonadherence can vary from suboptimal recovery to worsening SHCs [19, 20]. Importantly, mortality among individuals with CSCI who skip annual check-ups was found to be nearly four times higher than among those who attended regularly [21]. Given this context, it seems prudent to explore the prognostic value of self-reported health conditions and functioning for short- to medium-term survival using readily available data from the Swiss Community Survey linked to official vital statistics.

This study aimed to identify prognostic factors affecting survival within the SCI community, emphasizing measures of disease severity and self-reported data on functional independence (FI), SHCs, comorbidities, and psychological factors. Our ultimate goal was to determine whether self-reported ability to perform daily tasks, as well as self-reported health information, can provide an objective indicator of mortality, facilitate evidence-based, proactive, and individualized management, thereby enhancing the well-being of individuals with CSCI. This explorative study may contribute to the development of robust and validated instruments that can aid clinicians in identifying individuals at a higher mortality risk in the absence of recent clinical assessments or in the case of limited resources.

## Methods

### Study Population

This study utilized data from the first national community survey conducted in 2012 among individuals with CSCI [22], which is part of the population-based Swiss Spinal Cord Injury (SwiSCI) cohort study [23]. SwiSCI targets individuals aged 16 years and older who have been diagnosed with either traumatic or nontraumatic SCI and permanently reside in Switzerland. Individuals with congenital conditions leading to SCI, new injuries related to palliative care, neurodegenerative diseases, or Guillain-Barré syndrome were excluded. Individuals with CSCI who met the inclusion criteria outlined above were eligible to participate in the community study [22].

Vital status information up to the end of 2018 was acquired through probabilistic linkage with the Swiss National Cohort (SNC), utilizing date of birth, date of death (when available), geocoded address, age, and sex [9]. An in-depth description of the SNC can be found elsewhere [24]. For SwiSCI, the linkage dataset was based on its medical record study, which collected data on new SCI cases admitted to one of the Swiss specialized rehabilitation centers between 1967 and 2013. Of these cases, 85.5% were linked to the SNC [9]. This combined SwiSCI-SNC dataset partly overlaps with the participant database of the SwiSCI 2012 community survey, which did not include individuals who received their initial rehabilitation at the Balgrist Clinic in Zurich (one of the four active specialized rehabilitation centers in Switzerland).

A total of 3,144 individuals were eligible to participate in the SwiSCI 2012 community survey, and 1,549 individuals participated. Of the participants, 1,184 (76.4%) were included in the medical record study, and 1,028 (66.4%) were successfully linked to the SNC. Two linked cases were subsequently excluded because of an unknown etiology (traumatic vs. nontraumatic). In addition, individuals with a malignant tumor etiology (*n* = 15) were excluded because (1) there is evidence of substantially shortened survival compared to individuals with other nontraumatic etiologies [25] and (2) the small case number inhibits analysis as a separate group. CSCI is generally defined as the phase occurring at least 1-year post injury. The final study population included 1,011 cases (Fig. 1).

**Fig. 1.**
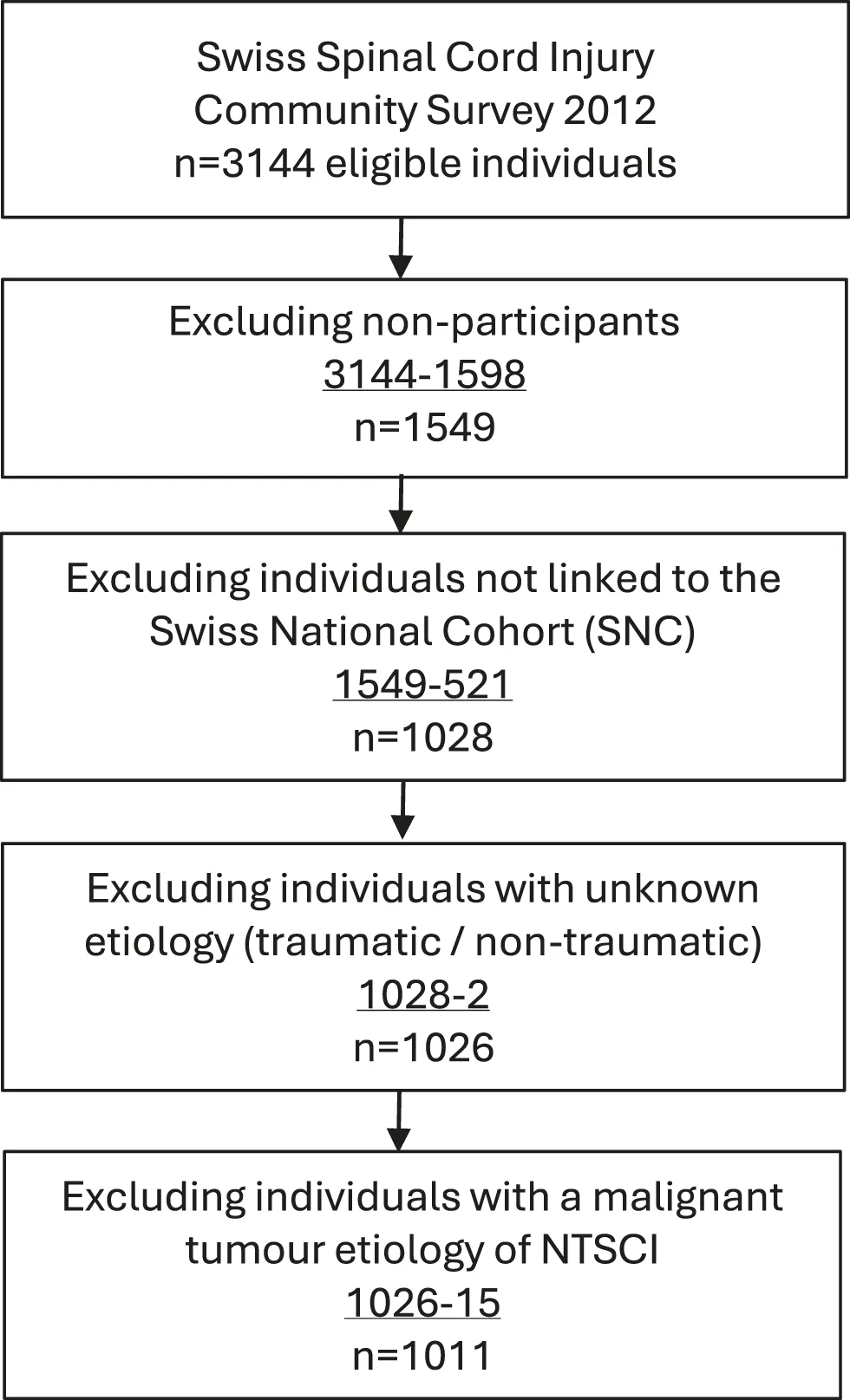
SwiSCI community survey sample.

### The SwiSCI Community Survey 2012 – Potential Predictors and Data Preparation

The SwiSCI Community Survey 2012 aimed to gather insights into participants’ lived experiences, including FI, physical health, and mental health [22, 23, 26]. The survey consisted of three successive modules, administered between September 2011 and March 2013. For this study, we utilized the first two modules: the Starter Module, which queried demographics and lesion characteristics, and the Basic Module, which focused on key domains of functioning. The survey questionnaires were made publicly available online [27].

Demographics and lesion characteristics were obtained from medical records [23]. This study included assessments of FI, SHC, comorbidities, health-related well-being, and QoL.

FI was measured using the self-report version of the Spinal Cord Independence Measure-III (SCIM-III-SR) [27, 28]. The SCIM-III is a comprehensive tool designed to assess the FI of individuals with SCI, evaluating three main areas: self-care, respiration and sphincter management; and mobility. SCIM-III is widely used in clinical settings to track rehabilitation progress and outcomes [29].

Data on whether individuals had diabetes, heart disease, cancer, or depression were collected using yes/no questions. The presence of 14 SHCs, such as respiratory problems and spasticity, was assessed using a categorical 4-level item: “not experienced/insignificant problem,” “mild or infrequent problem,” “moderate or occasional problem,” and “significant or chronic problem” [27]. Emotional well-being was evaluated using the corresponding 5-item subscale of the 36-Item Short Form Health Survey (SF-36) [30]. The SF-36 questionnaire measures health-related QoL using eight domains, including emotional well-being and mental health [30].

Additionally, QoL, satisfaction with health, and tiredness over the past few weeks were assessed using a single-item scale with five levels [27]. The QoL scale ranged from “very poor” to “very good,” satisfaction with health from “very satisfied” to “very dissatisfied,” and tiredness from “none” to “extreme.”

SCIM-III-SR subscale scores for self-care (SCIM-SC), SCIM-III-SR subscale scores for respiration and sphincter management (SCIM-RS), and SCIM-III-SR subscale scores for mobility (SCIM-M) were calculated by summing the scores for each individual item, as described by Fekete et al. [28]. The subscale scores range from 0 to 20 for the SCIM-SC and from 0 to 40 for both the SCIM-RS and SCIM-M, where 0 represents total dependence and 20 or 40 indicates total independence. Partially observed responses were treated as missing, and the lowest and highest possible values were calculated for later use.

The SF36 subscale for emotional well-being was calculated using the SF-36 Health Survey Manual [30]. If at least three items were completed, missing values were replaced with the average of the filled-in responses before calculating the subscale score [30]. If fewer than three items were completed, the lowest and highest possible values were calculated. The total score ranges from 0 to 100, with higher scores indicating better well-being. Age at injury and age at survey, as well as years since SCI, were categorized following the guidelines for reporting results from the International SCI Core Data Set for descriptive analyses, but were retained as continuous variables for model building [31].

### The Base Model

Demographic data and lesion characteristics were defined as base model predictors and were included in all potential prognostic models. These predictors included sex, age at the time of the survey, SCI etiology (traumatic or nontraumatic), lesion severity (incomplete, complete paraplegia or incomplete or complete tetraplegia), and years since injury at the time of the survey. This selection was based on the assumption that these basic parameters are typically known or can be easily obtained from patients along with other self-reported health information.

### Statistical Methods

The individual steps in model development are illustrated in the flowchart in Figure 2 and are detailed in the following sections. Flexible parametric survival models were used to model survival and calculate hazard ratios (HRs) with 95% confidence intervals (CIs) [32]. Time since survey participation served as the timescale for all models. The baseline hazard functions were modeled using restricted cubic splines with two internal knots placed at evenly distributed centiles of the uncensored log event times. All models were fitted on the log cumulative hazard scale. The number of knots and the model scale were selected to achieve the best model fit, as indicated by the Akaike Information Criterion (AIC) value. Model fit was tested in both “univariable” analyses (single predictors along with base model predictors, collectively referred to as “univariable” models) and in a complete model including all potential predictors, as well as before and after multiple imputation (Fig. 2).

**Fig. 2.**
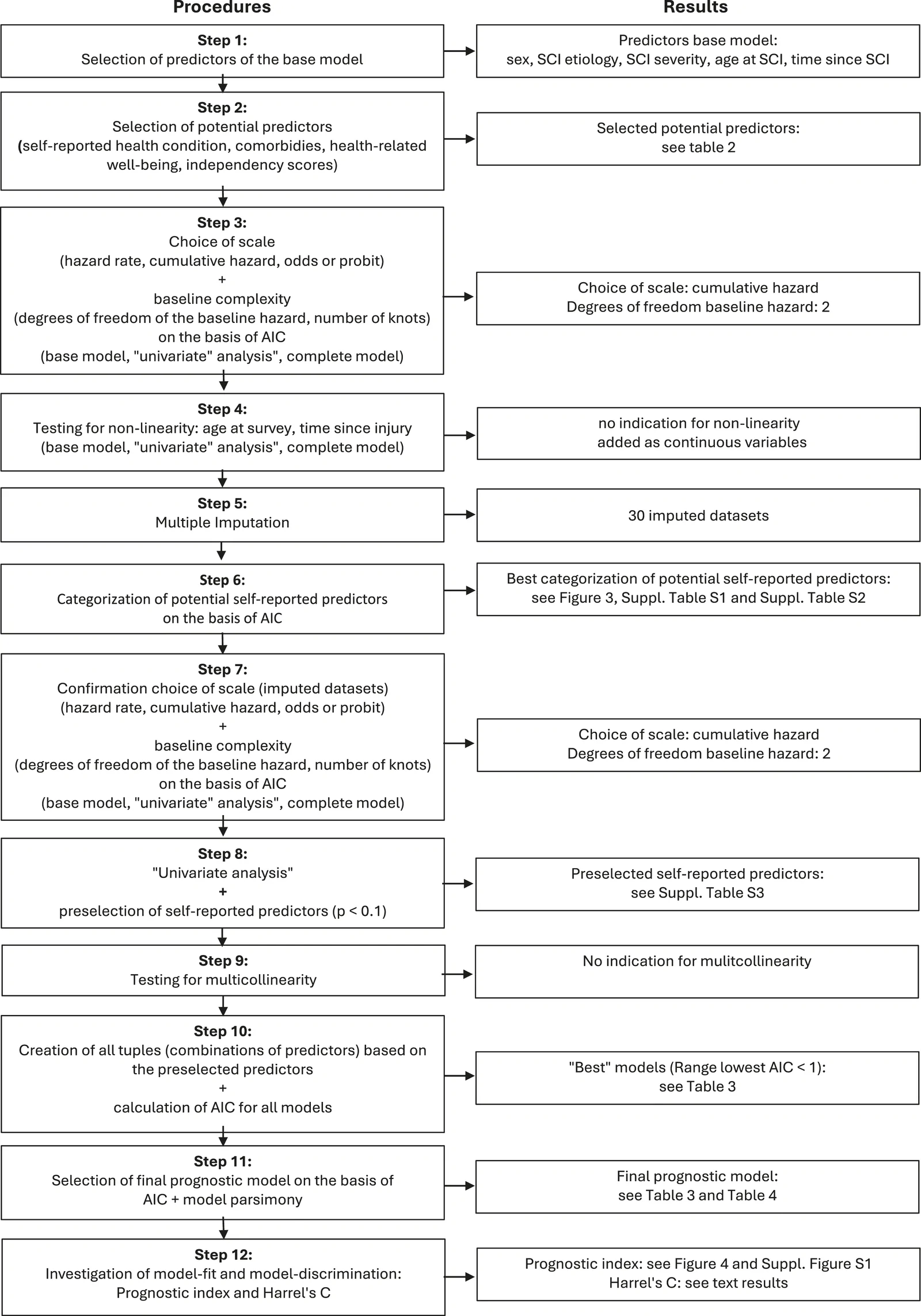
Flowchart model selection – procedures and results.

Possible nonlinear functional relationships between survival and the continuous variables of the base model (age at survey and time since injury) were investigated using fractional polynomials and their combinations, employing a backward elimination regression procedure in a complete-case analysis [33]. Nonlinearity was tested in both “univariable” models and the complete model (Fig. 2).

Missingness in the study dataset varied between 0% (demographic and lesion characteristics) and 20.9% (respiration and sphincter management score; SCIM-III-SR-RS) (Table 1, Suppl. Table S1, Suppl. Table S2; for all online suppl. material, see https://doi.org/10.1159/ned/adwag018). Missing data were addressed using multiple imputation with chained equations (m = 30). The imputation model included all potential predictors (variables of the base model plus all variables listed in Table 2), the Nelson-Aalen estimator of cumulative hazards, and the event (death)/censoring variable [34]. Binary comorbidity information was imputed using logistic regression. Multilevel response items, such as SHCs and QoL, were imputed using ordered logistic regression. The SCIM-III-SR and SF-36 were imputed using the interval regression method to account for partially observed responses. All subsequent analyses were conducted separately for each imputed dataset, and the results were combined using Rubin's Rules [35]. The *p* values of test statistics were merged by averaging across multiple imputations [36].

**Table 1.** Demographic and lesion characteristics of SwiSCI community survey sample

	Alive		Deceased			Total	
	*n*	%	*n*	%		*n*	%
Sex							
Male	669	74.3	86		78.2	755	74.7
Female	232	25.7	24		21.8	256	25.3
Age at SCI diagnosis^a^							
<16 years	7	0.8	1		0.9	8	0.8
16–30 years	381	42.3	24		21.8	405	40.1
31–45 years	236	26.2	14		12.7	250	24.7
46–60 years	170	18.9	31		28.2	201	19.9
61–75 years	97	10.8	35		31.8	132	13.1
≥76 years	10	1.1	5		4.5	15	1.5
Age at time of survey^b^							
16–30 years	79	8.8	1		0.9	80	7.9
31–45 years	231	25.6	3		2.7	234	23.1
46–60 years	348	38.6	31		28.2	379	37.5
61–75 years	205	22.8	50		45.5	255	25.2
76+ years	38	4.2	25		22.7	63	6.2
SCI etiology							
traumatic SCI	758	84.1	78		70.9	836	82.7
Nontraumatic SCI	143	15.9	32		29.1	175	17.3
Lesion severity							
Incomplete paraplegia	352	39.1	39		35.5	391	38.7
Complete paraplegia	266	29.5	36		32.7	302	29.9
Incomplete tetraplegia	190	21.1	18		16.4	208	20.6
Complete tetraplegia	93	10.3	17		15.5	110	10.9
Years since injury at survey^c^							
1–5 years	267	29.6	25	22.7		292	28.9
6–10 years	187	20.8	24	21.8		211	20.9
11–15 years	124	13.8	17	15.5		141	13.9
16–20 years	86	9.5	8	7.3		94	9.3
21–25 years	88	9.8	7	6.4		95	9.4
26–30 years	67	7.4	10	9.1		77	7.6
31–35 years	44	4.9	10	9.1		54	5.3
36+ years	38	4.2	9	8.2		47	4.6
Length of follow-up^d^							
<1 years	1	0.1	13	11.8		14	1.4
1–2 years	10	1.0	28	25.5		37	3.7
3–4 years	27	3.0	17	15.5		44	4.4
5–6 years	401	44.5	36	32.7		437	43.2
6–7 years	463	51.4	16	14.6		479	47.4
Total	901	100.0	110	100.0		1,011	100.0

All variables listed above were fully observed, with no missing data. For eight participants who were alive, follow-up time was censored before the end of the study period due to loss to follow-up.

IQR, interquartile range.

^a^ Median: 36; IQR [23;52].

^b^ Median: 52; IQR [41;64].

^c^ Median: 11; IQR [5;21].

^d^ Median: 4; IQR [4;5].

**Table 2.** Overview of potential predictors – self-reported health conditions, comorbidities, health-related well-being, and independence scores of SwiSCI community survey sample

Potential predictors	Original scale	Included after “univariable” analyses	Included in final model
Secondary health conditions			
Sleep problem	Categorical, 4-level item	No	No
Injury caused by loss of sensation	Categorical, 4-level item	No	No
Pain severity	Categorical, 4-level item	Yes	No
Hypotension	Categorical, 4-level item	No	No
Circulatory problems	Categorical, 4-level item	Yes	No
Respiratory problems	Categorical, 4-level item	Yes	Yes
Bowel problems	Categorical, 4-level item	No	No
Bladder problems	Categorical, 4-level item	No	No
Urinary tract infection	Categorical, 4-level item	No	No
Heterotopic ossification	Categorical, 4-level item	No	No
Contractures	Categorical, 4-level item	Yes	Yes
Spasticity	Categorical, 4-level item	No	No
Dysreflexia	Categorical, 4-level item	No	No
Pressure sores	Categorical, 4-level item	No	No
Comorbidities			No
Depression	Binary (no/yes)	Yes	No
Diabetes	Binary (no/yes)	Yes	No
Heart disease	Binary (no/yes)	Yes	No
Cancer	Binary (no/yes)	Yes	Yes
Health-related well-being			No
Tiredness	Categorical, 5-level item	Yes	No
Satisfaction with health	Categorical, 5-level item	Yes	No
Quality of life	Categorical, 4-level item	Yes	No
Emotional well-being (SF-36-Score)	Continuous score (0–100)	No	No
Functional independence (SCIM-III-SR)			No
Self-care	Continuous score (0–20)	Yes	Yes
Respiration and sphincter management	Continuous score (0–40)	Yes	Yes
Mobility	Continuous score (0–40)	Yes	No

To enhance the statistical power of our analysis and avoid categories with low frequencies, we reduced the number of levels of multi-categorical variables by combining adjacent levels (Fig. 3). First, we created a new variable for each possible combination of adjacent levels that decreased the number of levels by at least one. Next, we compared the univariable model fit associated with each of these new variables and the original variable. Dichotomization produced the best model fit for all the multi-categorical variables. The multi-categorical level combinations that provided the best model fit are presented in online Supplementary Table S1. All binary variables were left unchanged.

**Fig. 3.**
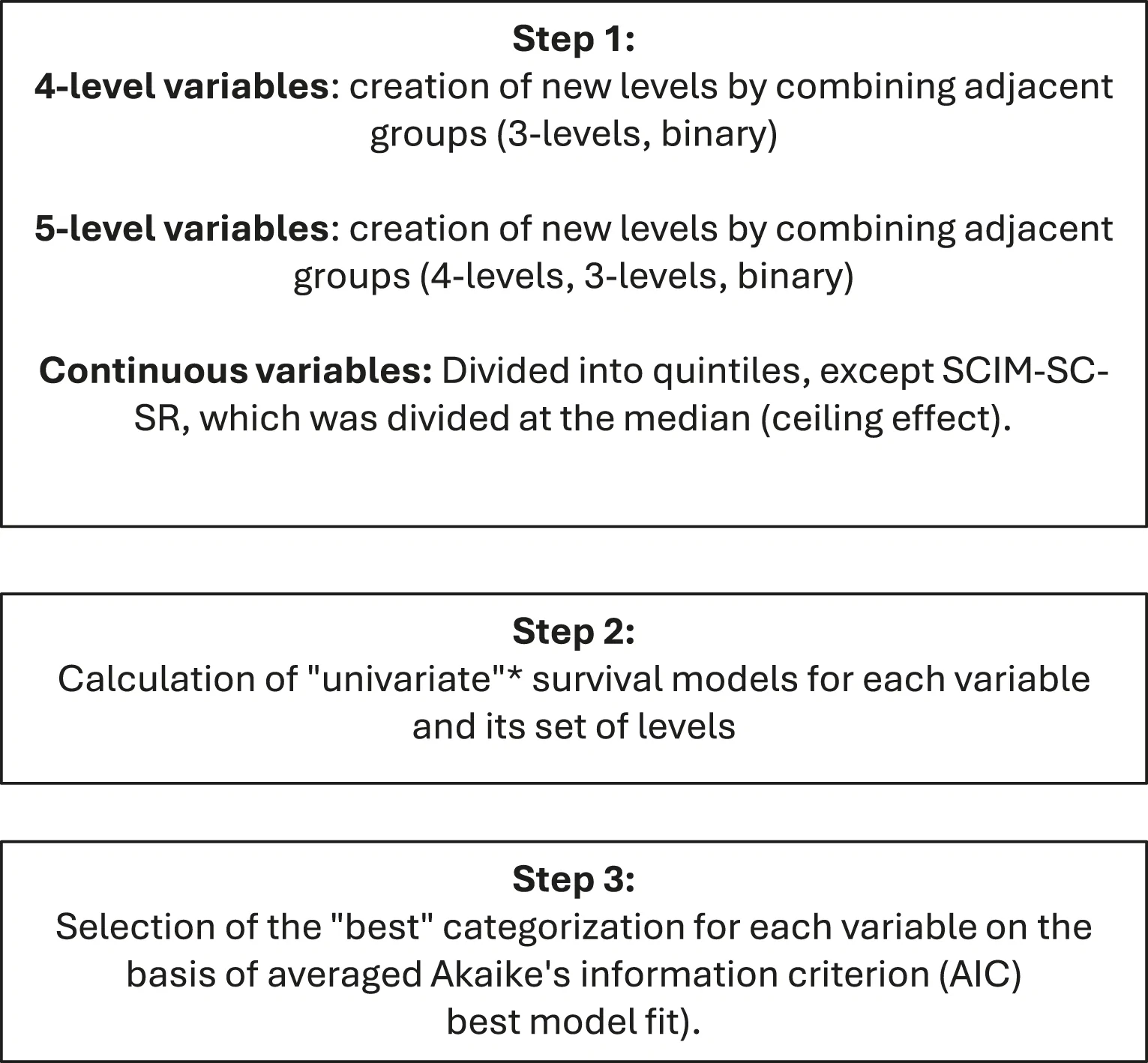
Categorization of potential predictors. *Each potential predictor was evaluated in addition to the base model covariates: sex, etiology of SCI, severity of SCI, age at injury, and time since injury.

The scores of SCIM-RS, SCIM-M, and emotional well-being were initially categorized into quintiles. Similar to other multi-categorical variables, the univariable model fit associated with all possible combinations of adjacent categories was compared to select the best model for further use. The best model fit was again achieved through dichotomization, which also proved superior to including variables as continuous predictors. SCIM-SC was categorized by its median owing to a strong ceiling effect.

As the next step in our analysis, we focused on selecting variables based on their *p* values from the univariable analysis, retaining only those with a *p* value <0.1 for inclusion in the multivariable modeling. Potential multicollinearity among all candidate predictors was assessed prior to multivariable modeling. Associations between continuous and binary predictors were examined using Pearson correlation coefficients, and associations involving the categorical variable lesion severity were assessed using Cramér’s V. The strongest correlation was observed between self-reported QoL and perceived quality of health (*r* = 0.56), representing a moderate association. Accordingly, all variables were retained for multivariable model selection.

For the retained variables, we created all possible tuples, estimated the model fit for each predictor combination, and selected the models with the best fit. Interaction terms and time-varying effects were not examined due to the limited number of events and the absence of prespecified clinical hypotheses. To minimize model complexity and overfitting, the analysis focused on main effects.

AIC values differing by less than 1 were considered to indicate an equal fit. The final model was selected based on the AIC and parsimony. The significance of the contribution of each predictor was additionally assessed using likelihood-ratio tests. The discriminatory power of the final model was assessed using Harrell’s C concordance index. Calibration of the final model was assessed quantitatively using the calibration slope and graphically by comparing model-predicted survival curves with Kaplan-Meier estimates across four prognostic index groups. The prognostic index graph illustrates the probability of death based on the patient characteristics included in the model. Additionally, model-based survival predictions and Kaplan-Meier estimates were graphically compared for each predictor, based on variable levels for categorical predictors and variable distributions for continuous predictors.

All statistical analyses were performed using Stata 18.0 (Stata Corporation, TX, USA). The study results are reported in accordance with the STROBE (Strengthening the Reporting of Observational Studies in Epidemiology) guidelines [37]. The completed STROBE checklist is provided as online supplementary material.

## Results

### Characteristics of the Study Population

The demographic and lesion characteristics of the SwiSCI community survey sample (N = 1,011) are presented in Table 1. The sample was primarily male (74.7%), had traumatic SCI (82.7%), and an incomplete paraplegic lesion (39.1%). The median age at the time of the survey was 52 years (IQR: 41–46), with a median time since injury of 11 years (IQR: 5–21). Participants were followed up for up to 7 years, contributing 6,557 cumulative person-years at risk, during which 110 (10.9%) died before the end of the study period. Survey results for all parameters of interest are presented in online Supplemental Tables S1 and S2, along with the frequencies of originally missing values.

### Linearity of Continuous Variables

Neither the base model nor the complete model showed an improved fit when considering potential nonlinear relationships of the two continuous variables: age at the time of the survey and time since injury. Consequently, both variables were included as linear predictors in all models.

### “Univariable” predictor selection

Based on “univariable” analyses, 14 of 25 predictors were selected for multivariable model selection. These included all three SCIM dimensions; four SHCs, including pain severity, circulatory problems, respiratory problems, and contractures; four comorbidities, including diabetes, heart disease, cancer, and depression; and three health-related measures of well-being, including tiredness, satisfaction with health, and QoL (Table 2 and online Suppl. Table S3).

### “Multivariable” predictor selection

Table 3 presents the “best models” that showed equally good fit. In addition to the base model predictors, the number of included predictors varied from five to seven. Respiratory problems, cancer, and SCIM-RS were selected as predictors in all eight models. Based on the predefined criteria (AIC and parsimony), the final model included the following predictors: respiratory problems, contractures, cancer, SCIM-RS, and SCIM-SC, along with the base model predictors (Table 3). Of the base model predictors, only age at the time of survey and sex significantly contributed to the final model (p_LRT_<0.05). HRs and corresponding CIs of the final model are presented in Table 4.

**Table 3.** Best fit models according to Akaike Information Criterion (AIC)

Best fit models according to AIC	*N*, predictors self-report	AIC
Respiratory problems + cancer + SCIM-RS + contractures + heart disease + SCIM-SC + base model	6	752.10
Respiratory problems + cancer + SCIM-RS + contractures + heart disease + SCIM-SC + SCIM-M + base model	7	752.16
Respiratory problems + cancer + SCIM-RS + contractures + SCIM-SC + base model	5	752.22
Respiratory problems + cancer + SCIM-RS + contractures + heart disease + SCIM-M-SR + base model	6	752.55
Respiratory problems + cancer + SCIM-RS + contractures + SCIM-SC + SCIM-M + base model	6	752.58
Respiratory problems + cancer + SCIM-RS + heart disease + SCIM-SC + base model	5	752.71
Respiratory problems + cancer + SCIM-RS + contractures + SCIM-M + base model	5	753.03
Respiratory problems + cancer + SCIM-RS + heart disease + SCIM-SC + quality of health + base model	6	753.06
Base model: sex, SCI etiology, SCI severity, age at survey, time since SCI	5	783.76

Green shadowed line: selected final prognostic model.

SCIM-III, Spinal Cord Independence Measure (Version III); SCIM-RS, SCIM-respiratory and sphincter management self-report version; SCIM-SC, SCIM-self care self-report version; SCIM-M, SCIM-mobility self-report version.

**Table 4.** Final flexible parametric survival model

Predictor	HR (95% CI)	SE	*p* value
		log-scale	
Respiratory problems			0.002
Not experienced/mild (ref.)	1		
Moderate/significant	2.06 (1.29–3.29)	1.29	
Contractures			0.125
No (ref.)	1		
Yes	1.42 (0.91–2.23)	0.91	
Cancer			0.084
No (ref.)	1		
Yes	1.86 (0.92–3.77)	0.92	
SCIM-SC			0.038
≥Median score (ref.)	1		
<Median score	1.66 (1.03–2.68)	1.03	
SCIM-RS			0.012
Quintile 2–5 (ref.)	1		
Quintile 1	1.77 (1.13–2.78)	1.13	
Sex			0.001
Female (ref.)	1		
Male	2.15 (1.34–3.43)	1.34	
Lesion etiology			0.213
Traumatic SCI (ref.)	1		
Nontraumatic SCI	1.34 (0.85–2.11)	0.85	
Lesion severity			0.449
Incomplete paraplegia (ref.)	1		
Complete paraplegia	1.21 (0.73–2.02)	0.73	
Incomplete tetraplegia	0.79 (0.46–1.37)	0.46	
Complete tetraplegia	1.21 (0.56–2.62)	0.56	
Age at survey, years	1.09 (1.07–1.10)	1.07	0.000
Time since SCI at survey, years	0.99 (0.98–1.01)	0.98	0.536

Harrell’s C-index: 0.849.

### Model Performance

Harrell’s C value for the final model was 0.849. Model-predicted survival closely tracked the observed Kaplan-Meier estimates within each group, suggesting good calibration (Fig. 4). The apparent calibration slope was 0.99 (95% CI 0.84–1.13), indicating no evidence of substantial overfitting in the development dataset. The survival probabilities for the different prognostic groups at 7 years after the survey ranged from 98.9% to 66.8%. Online Supplemental Table S4 provides examples of specific combinations of patient characteristics that led to the classification of each prognostic group.

**Fig. 4.**
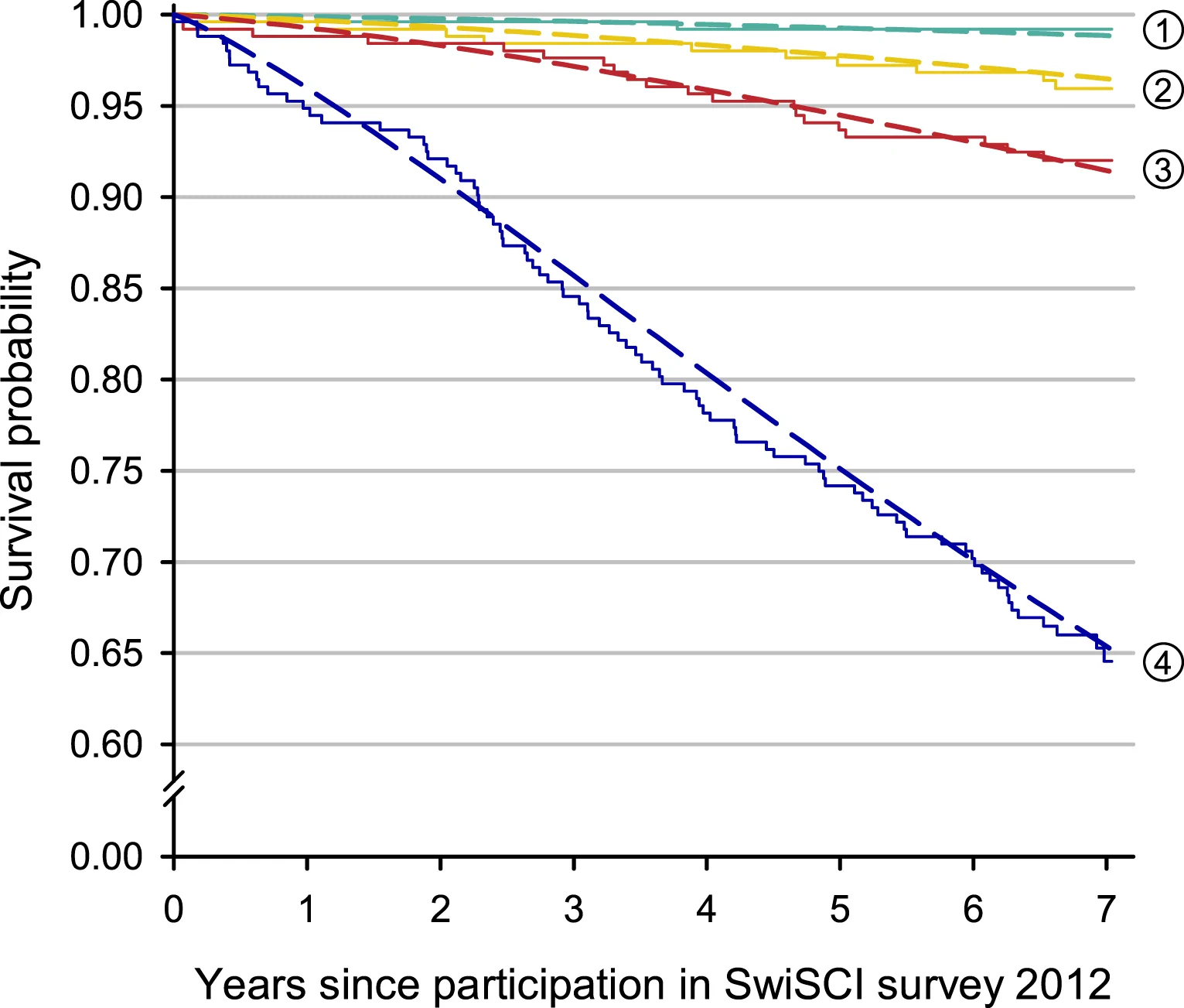
Prognostic index of the final prognostic model. Dashed lines indicate model predictions; solid lines indicate Kaplan-Meier estimates. The numbers 1–4 correspond to the risk groups, as defined by the prognostic index (1: lowest risk of death; 4: highest risk of death).

At the predictor level, model-based predictions and the corresponding Kaplan-Meier curves also indicated a good model fit (Suppl. Fig. S1). Age at survey showed the strongest discriminatory capability.

## Discussion

### Summary of Main Findings

The final prognostic model demonstrated clear discrimination among the four prognostic groups and good fit to the observed survival over a period of 7 years post survey in individuals with CSCI. The model incorporated self-reported information on respiratory problems, contractures and cancer, as well as the FI measures of self-care, and respiratory and sphincter management, along with basic information such as age, sex, and lesion severity. Alternative models with similarly good fit identified self-reported status regarding heart disease, mobility, and QoL as potential additional or alternative candidates with predictive value in multivariable prognostic models.

### Discussion in the Context of the Literature

Numerous studies have identified key predictors of survival following SCI. Commonly reported predictors include sex, age at injury, neurological level, extent of lesion, and year of injury [4, 5]. Additionally, the development of SCI-related health conditions, the need for mechanical ventilation, and the lack of access to specialized rehabilitation services have been consistently found to be negatively associated with survival [4, 5]. Other predictors reported in the literature include education, health behavior, quality of care, pre-injury health status, employment status, income, psychological factors, QoL, and specific SCI-related factors, such as neurogenic bowel dysfunction and frequent infections [5]. However, studies on SCI survival typically utilized time since injury (e.g., [38, 39]), time since admission to rehabilitation (e.g., [25, 40]) or time since 1-year survival past injury (e.g., [41] as key time points. Nevertheless, similar to our study, a body of research has investigated factors associated with residual survival in individuals with CSCI based on prevalent cases, most of which depend on or exclusively rely on self-reported information. The majority of these studies originated from a research group at the Medical University of South Carolina in the USA, based on a cohort of traumatic SCI cases identified at one specialized center in the Southeastern US The research group identified a broad range of uni- and multivariable predictors beyond demographic and lesion characteristics, which can be classified as follows [42]: (a) psychological and socio-environmental factors (e.g., neuroticism-anxiety [43]; income [44]; social support [44]), (b) behavioral factors (e.g., smoking and binge drinking [45]), and (c) secondary conditions and other health factors (e.g., chronic pressure ulcers; amputations; major depression [46]). However, in prediction modelling, the ultimate selection of predictors is significantly influenced by the candidate predictors that are considered at the initial stage. For this study, potential predictors were prespecified based on prior evidence, clinical plausibility, and the availability of data at community-level including self-reported data on FI, SHCs, comorbidities, and emotional well-being.

### Demographic and Lesion Characteristics

Sex and age at the time of the survey were significant independent predictors, with males exhibiting increased mortality rates, as consistently reported in other studies [4, 5]. As expected, age at the time of the survey was the strongest predictor, with a hazard ratio of 2.3 for each 10-year increase. Lesion severity, lesion etiology, and time since injury – all of which were included in the base model – were not predictive in the final model or in any of the models with a comparably good fit. Mortality rates are particularly divergent in the first years after the injury, with higher mortality rates in individuals with more severe injuries; however, they become more similar and stabilize over time if appropriate medical care and rehabilitation are provided [25, 38]. Notably, our study included individuals with CSCI, with a median time since injury of 11 years. Additionally, the final model included two dimensions of FI. Lesion severity and FI are not independent of each other. In our study, lesion severity was moderately associated with all three measures of FI (Cramér’s V ranging from 0.32 for SCIM-SC to 0.49 for SCIM-M). Further analyses demonstrated that lesion severity was a significant predictor of mortality in The base model; however, this significance was also observed when lesion severity was modeled jointly with FI (results not presented but available upon request).

### Comorbidities

This study investigated four common comorbidities as potential predictors and revealed self-reported prevalences of 15.6% for depression, 9.2% for heart disease, 10% for diabetes, and 3% for cancer. Notably, despite its relatively low prevalence in the 2012 population survey, cancer was included as a predictive factor in all eight superior models, each exhibiting an equally good fit to the observed data. Importantly, CSCI cases with a malignant etiology, known for their poor prognosis [25], were excluded from the analyses due to the limited number of cases in the dataset (*n* = 15). It is well known from the general population that survival outcomes vary significantly based on the cancer site and stage at diagnosis [47, 48]. However, in this study, the mere presence of a cancer diagnosis emerged as a highly predictive factor for increased mortality within the CSCI community. This underscores the need for further investigation into the implications of cancer comorbidities in this population.

A self-reported history of heart disease was included in five out of the eight equally well-performing models. One of these models was as parsimonious as the final chosen model, with the only difference being that heart disease replaced contractures. This model can be considered fully equivalent in terms of both model fit and parsimony, which were the predefined criteria for model selection. Therefore, the selection of either model would have been equally justified.

### Secondary Health Conditions

Of the 14 SHCs included in the study, only four demonstrated predictive value in the initial analyses, of which two remained in the final model: respiratory problems and contractures, both categorized by severity. Specifically, the final model encompassed moderate-to-severe respiratory issues and severe or chronic contractures, as opposed to less severe forms or the absence of the condition. Respiratory problems were included in all eight equally well-performing models, whereas contractures were selected as a predictor in six out of the eight models. Respiratory problems have been repeatedly reported as a major cause of death in people with CSCI, including findings from a Swiss study [4, 5, 9]. In contrast, contractures do not directly cause death but can lead to death via immobility-related complications, such as severe respiratory failure, embolism, pressure sores, and subsequent infections. To the best of our knowledge, this is the first study to include contractures as a potential predictor of survival following SCI. Based on the results outlined above, it seems advisable to consider contractures in future investigations concerning survival after CSCI.

### Functional Independence

Each of the three dimensions of FI measured by the self-report version of the SCIM-III instrument was selected in at least three of the equally well-performing models (SCIM-RS: 8/8, SCIM-SC: 6/8, SCIM-M: 3/8). Based on the model selection criteria, the final model included SCIM-RS and SCIM-SC. FI has been repeatedly identified as a predictor of survival in individuals with SCI [49, 50]. However, to the best of our knowledge, none of the previous studies have investigated the predictive value of self-reported FI in prevalent CSCI cases. The results underscore the importance of FI in estimating residual survival in individuals with CSCI and provide further support for the validity of the SCIM-III-SR as a measurement instrument for FI in individuals with SCI.

### Health-Related Well-Being

Measures of health-related well-being included the level of tiredness, satisfaction with health, QoL, and emotional well-being. Tiredness, satisfaction with health, and QoL showed significant associations in the univariable analyses that included the variables from the base model. However, only QoL emerged as a relevant predictor in one of the eight multivariable models, demonstrating an equally good fit. QoL has been shown to be predictive of survival in both the general population and individuals with CSCI [51, 52]. Our study suggests that measures of health-related well-being should be considered potential predictors of survival in future research, particularly when detailed information on comorbidities, SHCs, and FI is lacking.

### Strengths and Limitations

To our knowledge, this is the first study specifically aiming at investigating the prognostic value of self-reported health information for survival in individuals with CSCI in a population-based community setting. Both model development and the selection of predictors followed a predefined systematic approach, resulting in face validity for the prognostic value of self-reported health information within the CSCI community. The study included a wide range of potential predictors, such as FI, SHCs, comorbidities, and measures of health-related well-being, in addition to patient and lesion characteristics. We consider it an additional strength that the study includes all available prevalent cases, enabling the prediction of residual survival for those who survived until the survey based on recent (self-reported) health information. Furthermore, previous studies on this topic have generally been limited to traumatic CSCI. Additionally, this study provides a prognostic survival index to differentiate between risk groups, which has not been previously presented in similar studies.

This study also has certain limitations that need to be acknowledged when interpreting the findings and considering future research. The findings may not be generalizable to the entire Swiss SCI population. Approximately 50% of potential participants declined to complete the survey. Although a comprehensive investigation of the available demographic and lesion characteristics of responders and nonresponders of the community survey did not indicate evidence of bias, it is possible that other unmeasured characteristics may have influenced study inclusion and, consequently, the prognostic index of survival [22]. Moreover, among the participants, approximately two-thirds were linked to the SNC and were included in the study. However, the impartial linkage procedure does not offer a clear theoretical basis to suggest that linkage failure would be selectively affected by survival differentials.

For our study, vital status updates were only available until the end of 2018, as the census-based SNC is no longer updated by the SFSO beyond that year. This limited the analyses to a follow-up period of 7 years, and the derived prognostic index of survival may not be attuned to the most recent calendar years. According to expert opinions of the clinicians involved in this study, the management of CSCI in Switzerland remained largely unchanged between the analyzed period and the more recent calendar period. This is in line with literature evidence regarding CSCI management in Switzerland or other high-income countries over the recent decades. Nevertheless, secular trends in healthcare delivery or shifts in the health characteristics of the SCI population could affect the model’s performance in more recent years. Finally, our evaluation of prognostic factors did not include behavioral factors such as smoking or alcohol consumption, potentially protective factors such as adherence to follow-up care, or other relevant predictors including socioeconomic status, medication use, infection history, and pressure ulcer severity, as these variables were not available in the dataset. [42]. The omission of these factors may have limited the comprehensiveness of risk stratification.

Several statistical considerations must also be taken into account when evaluating the credibility of the derived prognostic index. Although the study is based on a comparably large number of individuals, resulting in more than 6,500 person-years at risk, the majority of participants survived beyond the follow-up period, leading to almost 90% of cases being censored. In survival analysis, statistical power is directly related to the number of observed events rather than the total sample size, which may explain the relatively large uncertainty of the coefficient estimates in our study [22]. A further limitation concerns the number of events relative to the number of model parameters. Although the final model was selected using AIC to promote parsimony, 110 deaths resulted in approximately 9 events per parameter, which is close to the commonly recommended threshold. This may increase the risk of overfitting and optimism in model performance. In the absence of formal internal validation, some degree of optimism in the reported discrimination cannot be excluded.

Another limitation concerns the dichotomization of several continuous predictors using data-driven cutoffs. While this approach may enhance interpretability and simplify clinical application, it can lead to loss of information and may improve apparent model fit in the development sample at the expense of generalizability of the model. Consequently, the stability and transportability of these cutoff values should be interpreted with caution and require confirmation in future validation studies.

Additionally, as in the referenced studies, the prognostic model did not undergo internal or external validation. Given the limited number of events relative to the number of estimated parameters, we considered the model exploratory and refrained from conducting resampling-based internal validation, as such procedures may yield unstable estimates in small samples. Future research using updated vital statistics and larger numbers of events is necessary to allow robust internal validation and external replication.

An important consideration is whether the predictors identified in our model may represent potential targets for intervention. However, inclusion in a prognostic model does not imply that modifying a predictor would necessarily improve outcomes, as establishing modifiable determinants of survival requires a causal framework and an appropriate study design. Nevertheless, some domains retained in our model, such as FI, contractures, and respiratory problems, may represent clinically relevant and potentially modifiable aspects of care. These findings may therefore inform future research evaluating whether targeted interventions in these areas can influence medium- to long-term survival in individuals with CSCI.

## Conclusion

Self-reported FI and health conditions, assessed years after injury, provide valuable prognostic information for the survival of prevalent cases with CSCI. However, internal and external validation of the existing models is required before clinical implementation can be considered.

## Acknowledgments

We thank the SwiSCI collaborating institutions, namely the patient organization Swiss Paraplegics-Association, Nottwil; the specialized rehabilitation centers Clinique Romande de Réadaptation, Sion; REHAB Basel, Basel; Spinal Cord Injury Center Balgrist University Hospital, Zurich; Swiss Paraplegic Centre, Nottwil; the academic institutions Competence Center for Rehabilitation Engineering and Science (RESC) ETH Zurich, Zurich; Faculty of Health Sciences and Medicine University of Lucerne, Lucerne; Institute of Social and Preventive Medicine (ISPM) University of Bern, Bern; the SCI Advocacy Board (Individuals living with SCI) as well as Swiss Paraplegic Research (SwiSCI host institution), Nottwil. We thank the Federal Statistical Office (FSO) for providing mortality and census data and for the support which made the Swiss National Cohort and this study possible. This work was supported by the Swiss National Science Foundation (grant nos. 3347CO-108806, 33CS30_134273 and 33CS30_148415). The members of the Swiss National Cohort Scientific Board are Marcel Zwahlen (University of Berne), Matthias Egger (University of Berne), Viktor von Wyl (University of Zurich), Oliver Hämmig (University of Zurich), Murielle Bochud (University of Lausanne), Martin Röösli (University of Basel), and Markus Schwyn (Federal Statistical Office).

## Statement of Ethics

The SwiSCI cohort study was approved by the responsible ethics committees of Luzern (reference number: EKLU 1008), Basel-Stadt (EKBB 37/11), Valais (CCVEM 015/11), and Zürich (EKZH 2012–0049). Data Linkage with vital status information was approved via amendments to these ethical approvals. Ethical approval for the SwiSCI Survey 2012 was granted by the Ethics Committees of Lucerne (EKLU 11042), Basel-Stadt (EKBB 306/11), and Valais (CCVEM 042/11). Informed consent to participate in the study was obtained from all subjects prior to data collection and was documented with a signed consent form.

## Conflict of Interest Statement

The authors have no conflicts of interest to declare.

## Funding Sources

The Swiss Federal Statistical Office provided mortality and census data and support, which made this study possible. This study has been financed in the framework of the SwiSCI cohort study (www.swisci.ch), supported by the Swiss Paraplegic Foundation. The funder had no role in the design, data collection, data analysis, and reporting of this study.

## Author Contributions

Study conception, design, and statistical analysis: A.F., A.B., and M.W.G.B.; interpretation of the data: A.F., A.B., I.E.H., M.H., M.S., M.W.G.B., and X.J.; drafting the manuscript: A.F.; critical revision of the manuscript: A.F., A.B., I.E.H., M.H., M.S., M.W.G.B., and X.J. All authors have read and agreed to the published version of the manuscript.

## Data Availability

For the SNC: Individual data from different data sets were used for the construction of the SNC. All these data are the property of the Federal Statistical Office (FSO) and can only be made available by legal agreements with the FSO. This also applies to derivatives such as the analysis files used for this study. However, after approval of the SNC Scientific Board, a specific SNC module contract with the FSO would allow researchers to receive analysis files for replication of the analysis. For SwiSCI: Owing to our commitment to SwiSCI study participants and their privacy, data sets generated during the current study are not made publicly available. The SwiSCI study center requires, on behalf of the SwiSCI Study Group, contact prior to any planned data usage (contact@swisci.ch).

## References

[1] Budd MA , Gater DR Jr, Channell I. Psychosocial consequences of spinal cord injury: a narrative review. J Pers Med. 2022;12(7):1178. 10.3390/jpm12071178 PMC9320050 35887675

[2] Sargent L , Smitherman J, Sorenson M, Brown R, Starkweather A. Cognitive and physical impairment in spinal cord injury: a scoping review and call for new understanding. J Spinal Cord Med. 2023;46(3):343–66. 10.1080/10790268.2022.2134634 PMC10114976 36441038

[3] Graves LY , Keane KF, Taylor JY, Wang TF, Saligan L, Bogie KM. Subacute and chronic spinal cord injury: a scoping review of epigenetics and secondary health conditions. Epigenet Insights. 2023;16:25168657231205679. 10.1177/25168657231205679 PMC10612389 37900668

[4] van den Berg ME , Castellote JM, de Pedro-Cuesta J, Mahillo-Fernandez I. Survival after spinal cord injury: a systematic review. J Neurotrauma. 2010;27(8):1517–28. 10.1089/neu.2009.1138 20486810

[5] Zadra A , Bruni S, DE Tanti A, Saviola D, Ciavarella M, Cannavo G, . Life expectancy and long-term survival after traumatic spinal cord injury: a systematic review. Eur J Phys Rehabil Med. 2024;60(5):822–31. 10.23736/S1973-9087.24.08462-4 PMC11559252 38842067

[6] Garshick E , Kelley A, Cohen SA, Garrison A, Tun CG, Gagnon D, . A prospective assessment of mortality in chronic spinal cord injury. Spinal Cord. 2005;43(7):408–16. 10.1038/sj.sc.3101729 PMC1298182 15711609

[7] DiPiro ND , Cao Y, Krause JS. A prospective study of health behaviors and risk of all-cause and cause-specific mortality after spinal cord injury. Spinal Cord. 2019;57(11):933–41. 10.1038/s41393-019-0298-9 31127197

[8] Krause JS , Carter RE, Pickelsimer EE, Wilson D. A prospective study of health and risk of mortality after spinal cord injury. Arch Phys Med Rehabil. 2008;89(8):1482–91. 10.1016/j.apmr.2007.11.062 PMC2651080 18674984

[9] Chamberlain JD , Buzzell A, Gmunder HP, Hug K, Jordan X, Moser A, . Comparison of all-cause and cause-specific mortality of persons with traumatic spinal cord injuries to the general Swiss population: results from a national cohort Study. Neuroepidemiology. 2019;52(3–4):205–13. 10.1159/000496976 30763935

[10] Bickenbach J , Batistella L, Gutenbrunner C, Middleton J, Post MW, InSCI, . The international spinal cord injury Survey: the way forward. Arch Phys Med Rehabil. 2020;101(12):2227–32. 10.1016/j.apmr.2020.06.011 32663478

[11] Cardile D , Calderone A, De Luca R, Corallo F, Quartarone A, Calabro RS. The quality of life in patients with spinal cord injury: assessment and rehabilitation. J Clin Med. 2024;13(6):1820. 10.3390/jcm13061820 PMC10971730 38542044

[12] Zhao H , Cole S, Omodior K, Valdivia DS. Ageing with spinal cord injury: a longitudinal study. Ageing Soc. 2025;45(6):1187–209.

[13] Hobbs WR , Fowler JH. Prediction of mortality using on-line, self-reported health data: empirical test of the RealAge score. PLoS One. 2014;9(1):e86385. 10.1371/journal.pone.0086385 PMC3895041 24466068

[14] Lorem G , Cook S, Leon DA, Emaus N, Schirmer H. Self-reported health as a predictor of mortality: a cohort study of its relation to other health measurements and observation time. Sci Rep. 2020;10(1):4886. 10.1038/s41598-020-61603-0 PMC7078209 32184429

[15] Seow H , Tanuseputro P, Barbera L, Earle C, Guthrie D, Isenberg S, . Development and validation of a prognostic survival model with patient-reported outcomes for patients with cancer. JAMA Netw Open. 2020;3(4):e201768. 10.1001/jamanetworkopen.2020.1768 PMC7113728 32236529

[16] Haddad TC , Maita KC, Inselman JW, Avila FR, Torres-Guzman RA, Coffey JD, . Patient satisfaction with a multisite, Multiregional Remote Patient Monitoring Program for acute and chronic condition management: Survey-Based analysis. J Med Internet Res. 2023;25:e44528. 10.2196/44528 PMC10415939 37343182

[17] Smith W , Colbert BM, Namouz T, Caven D, Ewing JA, Albano AW. Remote patient monitoring is associated with improved outcomes in hypertension: a large, retrospective, cohort analysis. Healthc (Basel). 2024;12(16):1583. 10.3390/healthcare12161583 PMC11353537 39201142

[18] Eriks-Hoogland IE , Muller L, Hirsch BDN, Studer L, Gemperli A, Anderson CE. Non-adherence to follow-up care in persons with spinal cord injury within 10 years after initial rehabilitation. J Rehabil Med. 2024;56:jrm41083. 10.2340/jrm.v56.41083 PMC11367676 39185548

[19] Eriks-Hoogland I , Muller L, Baumberger M, Huber B, Michel F, Belfrage C, . Evidence based clinical practice guideline for follow-up care in persons with spinal cord injury. Front Rehabil Sci. 2024;5:1371556. 10.3389/fresc.2024.1371556 PMC11417090 39314837

[20] Pan H , Zhu L, Xu J, Ding M, Lin H, Chen Y, . Bladder emptying strategies, active Follow-up adherence and barriers in post-discharge spinal cord injury individuals: a multiple Center cross-sectional Study. Patient Prefer Adherence. 2025;19:623–33. 10.2147/PPA.S509537 PMC11927570 40124964

[21] Chamberlain JD , Eriks-Hoogland IE, Hug K, Jordan X, Schubert M, Brinkhof MWG. Attrition from specialised rehabilitation associated with an elevated mortality risk: results from a vital status tracing study in Swiss spinal cord injured patients. BMJ Open. 2020;10(7):e035752. 10.1136/bmjopen-2019-035752 PMC7351285 32647022

[22] Brinkhof MW , Fekete C, Chamberlain JD, Post MW, Gemperli A, SwiSCI Study Group. Swiss national community survey on functioning after spinal cord injury: protocol, characteristics of participants and determinants of non-response. J Rehabil Med. 2016;48(2):120–30. 10.2340/16501977-2050 26926916

[23] Post MW , Brinkhof MW, von Elm E, Boldt C, Brach M, Fekete C, . Design of the Swiss spinal cord injury Cohort Study. Am J Phys Med Rehabil. 2011;90(11 Suppl 2):S5–16. 10.1097/PHM.0b013e318230fd41 21975676

[24] Spoerri A , Zwahlen M, Egger M, Bopp M. The Swiss National Cohort: a unique database for national and international researchers. Int J Public Health. 2010;55(4):239–42. 10.1007/s00038-010-0160-5 20556473

[25] Buzzell A , Chamberlain JD, Gmunder HP, Hug K, Jordan X, Schubert M, . Survival after non-traumatic spinal cord injury: evidence from a population-based rehabilitation cohort in Switzerland. Spinal Cord. 2019;57(4):267–75. 10.1038/s41393-018-0212-x 30413804

[26] Fekete C , Segerer W, Gemperli A, Brinkhof MW, SwiSCI Study Group. Participation rates, response bias and response behaviours in the community survey of the Swiss Spinal Cord Injury Cohort Study (SwiSCI). BMC Med Res Methodol. 2015;15:80. 10.1186/s12874-015-0076-0 PMC4599658 26450702

[27] Swiss spinal cord injury Cohort Study - Study Design Community Survey nottwil, Switzerland: swiss paraplegic research (SPF); [Available from: https://www.swisci.ch/en/research/study-design/community-survey/the-community-survey

[28] Fekete C , Eriks-Hoogland I, Baumberger M, Catz A, Itzkovich M, Luthi H, . Development and validation of a self-report version of the Spinal Cord Independence Measure (SCIM III). Spinal Cord. 2013;51(1):40–7. 10.1038/sc.2012.87 22890418

[29] Maritz R , Fellinghauer C, Brach M, Curt A, Gmunder HP, Hopfe M, . A rasch-based comparison of the functional Independence measure and spinal cord Independence measure for outcome and quality in the rehabilitation of persons with spinal cord injury. J Rehabil Med. 2022;54:jrm00262. 10.2340/jrm.v54.82 PMC8892304 35166364

[30] Ware JE , Snow KK, Kosinski M, Gandek B. SF-36 Health Survey manual and interpretation guide. Boston, Massachusetts: The Health Institute, New England Medical Center; 1993.

[31] DeVivo MJ , Biering-Sorensen F, New P, Chen Y, International Spinal Cord Injury Data Set. Standardization of data analysis and reporting of results from the International Spinal Cord Injury Core Data Set. Spinal Cord. 2011;49(5):596–9. 10.1038/sc.2010.172 21135863

[32] Lambert PC , Royston P. Further development of flexible parametric models for survival analysis. The Stata J Promoting Commun Stat Stata. 2009;9(2):265–90.

[33] Sauerbrei W , Meier-Hirmer C, Benner A, Royston P. Multivariable regression model building by using fractional polynomials: description of SAS, STATA and R programs. Comput Stat Data Anal. 2006;50(12):3464–85.

[34] White IR , Royston P. Imputing missing covariate values for the Cox model. Stat Med. 2009;28(15):1982–98. 10.1002/sim.3618 PMC2998703 19452569

[35] Rubin D . Multiple imputation for nonresponse in surveys. New jork John Wiley & Sons; 1987.

[36] Panken AM , Heymans MW. A simple pooling method for variable selection in multiply imputed datasets outperformed complex methods. BMC Med Res Methodol. 2022;22(1):214. 10.1186/s12874-022-01693-8 PMC9351113 35927610

[37] von Elm E , Altman DG, Egger M, Pocock SJ, Gotzsche PC, Vandenbroucke JP, . Strengthening the reporting of Observational Studies in Epidemiology (STROBE) statement: guidelines for reporting observational studies. BMJ. 2007;335(7624):806–8. 10.1136/bmj.39335.541782.AD PMC2034723 17947786

[38] Chamberlain JD , Gmunder HP, Hug K, Jordan X, Moser A, Schubert M, . Differential survival after traumatic spinal cord injury: evidence from a multi-center longitudinal cohort study in Switzerland. Spinal Cord. 2018;56(10):920–30. 10.1038/s41393-018-0163-2 29895883

[39] Lidal IB , Snekkevik H, Aamodt G, Hjeltnes N, Biering-Sorensen F, Stanghelle JK. Mortality after spinal cord injury in Norway. J Rehabil Med. 2007;39(2):145–51. 10.2340/16501977-0017 17351697

[40] DeVivo MJ , Chen Y, Wen H. Cause of death trends among persons with spinal cord injury in the United States: 1960-2017. Arch Phys Med Rehabil. 2022;103(4):634–41. 10.1016/j.apmr.2021.09.019 34800477

[41] Frankel HL , Coll JR, Charlifue SW, Whiteneck GG, Gardner BP, Jamous MA, . Long-term survival in spinal cord injury: a fifty year investigation. Spinal Cord. 1998;36(4):266–74. 10.1038/sj.sc.3100638 9589527

[42] Krause JS , Saunders LL, DiPiro ND, Reed KS. Theoretical risk and prevention model for secondary health conditions and mortality after SCI: 15 years of research. Top Spinal Cord Inj Rehabil. 2013;19(1):15–24. 10.1310/sci1901-15 PMC3584350 23459002

[43] Krause JS , Cao Y, DiPiro N. Psychological factors and risk of mortality after spinal cord injury. J Spinal Cord Med. 2020;43(5):667–75. 10.1080/10790268.2019.1690766 PMC7534322 31794353

[44] Krause JS , Carter RE. Risk of mortality after spinal cord injury: relationship with social support, education, and income. Spinal cord. 2009;47(8):592–6. 10.1038/sc.2009.15 PMC2722690 19255586

[45] Krause JS , Saunders LL. Risk of mortality and life expectancy after spinal cord injury: the role of health behaviors and participation. Top Spinal Cord Inj Rehabil. 2010;16(2):53–60. 10.1310/sci1602-53 PMC3181073 21960734

[46] Krause JS , Saunders LL. Health, secondary conditions, and life expectancy after spinal cord injury. Arch Phys Med Rehabil. 2011;92(11):1770–5. 10.1016/j.apmr.2011.05.024 PMC3385509 22032212

[47] McPhail S , Johnson S, Greenberg D, Peake M, Rous B. Stage at diagnosis and early mortality from cancer in England. Br J Cancer. 2015;112(Suppl 1):S108–15. 10.1038/bjc.2015.49 PMC4385983 25734389

[48] Minicozzi P , Innos K, Sanchez MJ, Trama A, Walsh PM, Marcos-Gragera R, . Quality analysis of population-based information on cancer stage at diagnosis across Europe, with presentation of stage-specific cancer survival estimates: a EUROCARE-5 study. Eur J Cancer. 2017;84:335–53. 10.1016/j.ejca.2017.07.015 28865261

[49] Cohen J , Marino R, Sacco P, Terrin N. Association between the functional independence measure following spinal cord injury and long-term outcomes. Spinal Cord. 2012;50(10):728–33. 10.1038/sc.2012.50 22641254

[50] Hatch B , Wood-Wentz C, Therneau T, Walker M, Payne J, Reeves R. Factors predictive of survival and estimated years of life lost in the decade following nontraumatic and traumatic spinal cord injury. Spinal Cord. 2017;55(6):540–4. 10.1038/sc.2016.182 28169294

[51] Cao Y , DiPiro ND, Krause JS. Longitudinal changes in employment, health, participation, and quality-of-life and the relationships with long-term survival after spinal cord injury. Spinal cord. 2023;61(8):430–5. 10.1038/s41393-023-00882-6 PMC10767623 36854965

[52] Phyo AZZ , Freak-Poli R, Craig H, Gasevic D, Stocks NP, Gonzalez-Chica DA, . Quality of life and mortality in the general population: a systematic review and meta-analysis. BMC Public Health. 2020;20(1):1596. 10.1186/s12889-020-09639-9 PMC7646076 33153441

